# An effective method for the identification and separation of *Anopheles minimus*, the primary malaria vector in Thailand, and its sister species *Anopheles harrisoni*, with a comparison of their mating behaviors

**DOI:** 10.1186/s13071-017-2035-6

**Published:** 2017-02-21

**Authors:** Kritsana Taai, Ralph E. Harbach, Kittipat Aupalee, Wichai Srisuka, Thippawan Yasanga, Yasushi Otsuka, Atiporn Saeung

**Affiliations:** 1 Department of Parasitology, Faculty of Medicine, Chiang Mai University, Chiang Mai, 50200 Thailand; 2 Department of Life Sciences, Natural History Museum, Cromwell Road, London, SW7 5BD UK; 3 Entomology Section, Queen Sirikit Botanic Garden, P.O. Box 7, Chiang Mai, 50180 Thailand; 4 Medical Science Research Equipment Center, Faculty of Medicine, Chiang Mai University, Chiang Mai, 50200 Thailand; 5 Research Center for the Pacific Islands, Kagoshima University, Kagoshima, 890-8580 Japan

**Keywords:** *Anopheles minimus*, *Anopheles harrisoni*, SEM, Antennal sensilla, Stenogamous behavior

## Abstract

**Background:**

Species of the *Anopheles minimus* complex are considered to be the primary vectors of malaria in South and Southeast Asia. Two species of the complex, *Anopheles minimus* and *Anopheles harrisoni*, occur in Thailand. They are sympatric and difficult to accurately distinguish based on morphological characters. The aim of this study was to investigate the potential of antennal sensory organs to distinguish these two species. Additionally, we investigated their ability to mate in cages of different sizes, as well as the possible mechanism(s) that evokes stenogamous behavior.

**Methods:**

Large sensilla coeloconica present on the antennae of females of *An. minimus* and *An. harrisoni* were counted under a conventional light microscope and various types of antennal sensilla were examined under a scanning electron microscope (SEM). Determinations of mating ability were carried out in 20 and 30 cm^3^ cages with a density resting surface (DRS) of 7.2. The insemination rate, frequency of clasper (gonocoxopodite) movement of the male genitalia during induced copulation and duration of mating of the two species were compared.

**Results:**

The mean numbers of large sensilla coeloconica on antennal flagellomeres 1–8 and the mean number of large sensilla coeloconica on each flagellum in *An. minimus* (26.25) and *An. harrisoni* (31.98) were significantly different. Females of both species bear five types of antennal sensilla: chaetica, trichodea, basiconica, coeloconica and ampullacea. Marked differences in the structure of the large sensilla coeloconica were observed between the two species. Furthermore, only *An. minimus* could copulate naturally in the small cages. The frequency of clasper movement in the stenogamous *An. minimus* was significantly higher than in *An. harrisoni*, but there was no difference in the duration of mating.

**Conclusions:**

To our knowledge, this study is the first to examine and discover the usefulness of large sensilla coeloconica on the antennae of females and the frequency of clasper movement in males for distinguishing the sibling species *An. minimus* and *An. harrisoni*. The discovery provides an effective and relatively inexpensive method for their identification. Additionally, the greater frequency of clasper movement of *An. minimus* might influence its ability to mate in small spaces.

## Background

Malaria is a life-threatening disease caused by plasmodial protozoa that are transmitted to people through the bites of infected female *Anopheles* mosquitoes. About 3.2 billion people are at risk of malaria [1]. In 2015, there were roughly 214 million malaria cases and an estimated 438,000 deaths due to the disease. Malaria is one of the most important parasitic diseases in Thailand and has long been one of the major causes of morbidity and mortality, especially along the international borders with Cambodia, Myanmar and Malaysia. To date, there are 472 formally recognized species of *Anopheles* worldwide [2]. Seventy-five formally named species occur in Thailand [3–5]. Some species of the *An. dirus* complex (*An. baimaii* and *An. dirus*), *An. minimus* complex (*An. minimus*) and the *An. maculatus* group (*An. maculatus*) are recognized as primary malaria vectors in the country.

The *An. minimus* complex (henceforth the Minimus Complex) belongs to the Minimus Subgroup within the Funestus Group of the Myzomyia Series [2, 6]. The Minimus Complex includes three species, *An. minimus* (formerly *minimus* species A) [7, 8], *An. harrisoni* (formerly *minimus* species C) [7, 9] and *An. yaeyamaensis* (formerly *minimus* species E) [10]. The last species is only known to occur on the Yaeyama and Miyako Islands located at the southern end of the Ryukyu Archipelago of Japan. *Anopheles minimus* occurs throughout Thailand whereas *An. harrisoni* is confined to western and northern areas, including Tak and Chiang Mai Provinces [3]. *Anopheles minimus* was incriminated as a primary vector of malaria in Thailand, whereas the vector status of *An. harrisoni* has not yet been determined. *Anopheles minimus* is mainly anthropophilic, endophagic and exophilic, whereas *An. harrisoni* has shown a greater tendency toward zoophily, exophagy and exophily [11, 12]. In addition, it has been shown that *An. minimus* can copulate successfully in 30 cm^3^ cages (stenogamous colony) [13–15].

*Anopheles minimus* has often been misidentified because the adult females exhibit overlapping morphological characters with other members of the Funestus Group, particularly *An. aconitus*, *An. pampanai* and *An. varuna* [14]. Sucharit et al. [16] noted that the absence of a humoral pale spot (HP) on the costa of the wing could distinguish *An. minimus* from *An. harrisoni*. However, the findings of Green et al. [7], Sharpe [17], Chen et al. [18], Sungvornyothrin et al. [19] and Cuong et al. [20] showed that presence of a humeral pale spot was not reliable for distinguishing the two species. Subsequently, isoenzymes (esterase and octanol dehydrogenase) were used to distinguish the two species in an area of sympatry [7], but this identification method requires fresh or frozen specimens; thus, molecular methods have been used to identify them. Allele-specific polymerase chain reaction (AS-PCR) is frequently used to distinguish the two species from other species of the Funestus Group [21, 22]. Analyses based on mitochondrial DNA fragments of *An. minimus*, such as the cytochrome *c* oxidase subunits 1 (*cox*1) and 2 (*cox*2), have been used for molecular identification, as well as for phylogenetic and population genetics studies, of species of the Minimus Subgroup [20, 23–25].

Researchers have attempted to find effective methods for the identification of *Anopheles* vectors based on morphological attributes, e.g. light microscopy of antennal sensilla [26] and scanning electron microscopy (SEM) of eggs and antennal sensilla [27–30]. However, no studies of the antennal sensilla of members of the Minimus Complex have been conducted. Hence, the purpose of this study was to examine and compare the antennal sensilla in females of *An. minimus* and *An. harrisoni* using light and scanning electron microscopy. The various types of sensilla borne on the antennae of the two species are described herein. In addition, we investigated the mating behavior of *An. minimus* (stenogamous) and *An. harrisoni* (eurygamous) by comparing the insemination rates, frequency of clasper (gonocoxopodite) movement of the male genitalia during induced copulation and duration of copulation of the two species.

## Methods

### Mosquitoes and species identification

Free mating (stenogamous) *An. minimus* were obtained from the Office of Disease Prevention and Control No. 1, Department of Disease Control, Ministry of Public Health, Chiang Mai Province, Thailand. Specimens of *An. harrisoni* were reared from wild-caught larvae collected in Ban Pu Tuey (village), Sai Yok District, Kanchanaburi Province, Thailand. Specimens and reared F_1_ progeny from individual females (see below) were identified to species using morphological keys for the *Anopheles* in Thailand [3]. Following morphological identification, molecular identifications were performed using the AS-PCR assay based on ITS2 rDNA sequences [22]. Genomic DNA was extracted from individual adult females using the DNeasy® Blood and Tissue Kit (Qiagen, Hilden, Germany) and isolated DNA was subjected to sequential PCR procedures. The ITS2 region was amplified using the universal forward primer ITS2A (5′-TGT GAA CTG CAG GAC ACA T-3′) and the specific reverse primers MIA (5′-CCC GTG CGA CTT GAC GA-3′ for *An. minimus*) and MIC (5′-GTT CAT TCA GCA ACA TCA GT-3′ for *An. harrisoni*). PCR was carried out using 25 μl volumes containing 0.5 U of *Taq* DNA polymerase, 1× *Taq* buffer, 2.0 mM of MgCl_2_, 0.2 mM of each dNTP, 0.25 μM of each primer and 1 μl of the extracted DNA. The amplification profile comprised initial denaturation at 94 °C for 2 min, 30 cycles at 94 °C for 30 s, 45 °C for 30 s and 72 °C for 40 s and a final extension at 72 °C for 5 min. The amplified products were electrophoresed on 1.5% agarose gel. In addition, PCR products were sequenced using the BigDye® Terminator Cycle Sequencing Kit and analyzed with the 3130 genetic analyzer for species confirmation.

### Mosquito rearing

Larvae of *An. minimus* and *An. harrisoni* were reared to adults using the procedure described by Choochote and Saeung [31]. Mosquitoes were colonized and maintained continuously for several generations at 27 ± 2 °C, 70–80% relative humidity and with illumination from a combination of natural daylight from a glass window and fluorescent lighting provided for approximately 12 h a day in an insectary of the Department of Parasitology, Chiang Mai University. Five days after emergence, meals of bovine blood acquired from a slaughterhouse were provided with an artificial membrane feeding method [31, 32].

### Light microscopy

Large sensilla coeloconica (peg organs in pits) on antennal flagella were observed using an Olympus BX53 compound microscope. These sensilla were counted and compared in 36 h post-emerged females. Females from each species were immersed in 10% potassium hydroxide and left for 30–45 min in a hot oven. After clearing, they were washed with 80% ethanol, their antennae were removed using an insect needle and the two antennae of each female were mounted together in Hoyer’s medium on a microscope slide. The large sensilla coeloconica borne on the left and right flagellum of 30 females of each species were counted (*n* = 60 flagella/species).

### Scanning electron microscopy

Thirty heads of 4- to 5-day-old females of each species were excised under a stereomicroscope and rinsed three times in phosphate buffer (pH 7.4) to remove surface debris. The heads were then dehydrated through an ethanol series of 35, 70, 80% (10 min, two changes) and 95% (15 min, two changes), followed by absolute ethanol (10 min, two changes). Following dehydration, they were dried in a critical point dryer. The antennae were carefully dissected from the head capsule under a stereomicroscope, as described by Hempolchom et al. [30]. Antennae were mounted on aluminum stubs with double-sided carbon adhesive tape and sputter-coated with gold. Sensilla were observed and photographed in a JEOL-JSM6610LV scanning electron microscope (JEOL, Japan).

### Mating of adult mosquitoes

Adult F_9_ mosquitoes of *An. minimus* and *An. harrisoni* were used to observe their mating ability in cages of two sizes [33]. Density resting surface (DRS) of the cages was calculated by dividing the vertical resting surface area (RS) (cm^2^) by the mosquito population density (D) [34]. Comparison of mating ability in 20 and 30 cm^3^ cages with DRS of 7.2 was carried out using various ratios of female/male mosquitoes. At a DRS of 7.2, female/male (total) cohorts of 89/133 (222) and 200/300 (500) were introduced into the 20 and 30 cm^3^ cages, respectively, where they remained for 1 week. Solutions of 10% sucrose and 5% multivitamin syrup were provided as nutrients [31]. Subsequently, mean insemination rate was calculated from dissection of 200 spermathecae (duplicate experiments, 100 spermathecae/experiment). Intact spermathecae were placed on microscope slides and examined for movement of the long thread-like spermatozoa under 100× magnification using the compound microscope mentioned above. Spermathecae with detected movement of spermatozoa were ruptured by placing a coverslip on the microscope slide, and the surrounding field was scanned for spermatozoa. The relative percentage of the volume of spermathecae containing spermatozoa was graded as 0 (fairly transparent or uninseminated spermatheca), 1+ (25% of area of coverslip with spermatozoa), 2+ (50% of area of coverslip with spermatozoa), 3+ (75% of area of coverslip with spermatozoa) and 4+ (100% of area − spermatheca full of spermatozoa) [26].

### Frequency of clasper movement and mating time

The procedures described by Wijit et al. [26] were used for measuring clasper movement and the duration of mating. The frequency of clasper movement during induced copulation and the duration of mating were determined using 5-day-old females and males of each species (*n* = 30/species). During induced copulation, females are grasped initially by the claspers of males and remain coupled for a period of time before sperm transfer, with movement of their claspers until separation. Two persons participated in this experiment. The frequency of clasper movement was observed and counted under a stereomicroscope by one person while the other person, the timekeeper, measured the duration of mating with the aid of an electric watch.

### Statistical analysis

The Chi-square test was used to determined insemination rates. The numbers of large sensilla coeloconica on female antennae, and frequency of clasper movement during induced copulation and mating times, were assessed by Student’s *t*-test. All data were analyzed using SPSS v. 20.0 for Windows (Chicago, SPSS Inc.). Statistical significance was set at *P* < 0.05.

## Results

### Molecular identification

**Fig. 1:**
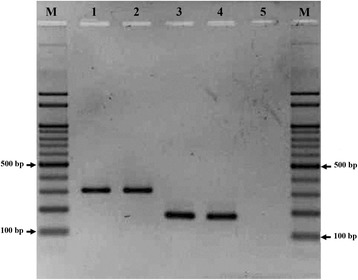
Gel of allele-specific PCR for identifying *An. minimus* and *An. harrisoni*. Lanes 1 and 2: *An. minimus* (310 bp); Lanes 3 and 4: *An. harrisoni* (180 bp); Lane M: 100 bp ladder

The AS-PCR successfully confirmed the morphological identification of *An. minimus* and *An. harrisoni*. PCR species-specific products were 310 bp and 180 bp for *An. minimus* and *An. harrisoni*, respectively (Fig. 1).

### General morphology of the antennae of females

**Fig. 2:**
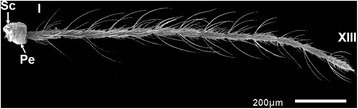
Scanning electron micrograph of the antenna of *An. minimus* (virtually identical in *An. harrisoni*). The scape (Sc), pedicel (Pe) and the first and thirteenth flagellomeres are labelled

**Fig. 3:**
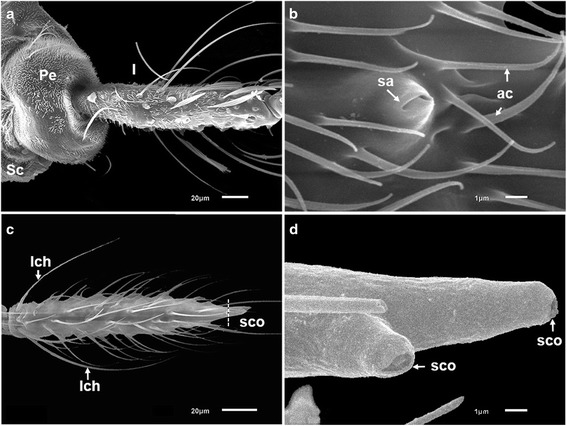
Scanning electron micrographs of flagellomere 1 and the tip of flagellomere 13 (representative of both *An. minimus* and *An. harrisoni*). **a** Scape (Sc), pedicel (Pe) and first flagellomere (I) densely covered with aculeae. **b** Higher magnification of sensillum ampullaceum (sa) surrounded by aculeae (ac). **c** Large sensilla chaetica (lch) at the base and small sensilla coeloconica (sco) at the tip of flagellomere 13. **d** Higher magnification of sensilla coeloconica (sco) at the tip of flagellomere 13

The antennae of females of *An. minimus* and *An. harrisoni* are morphologically similar (Fig. 2). The antennae of mosquitoes consist of a basal scape, a pedicel and a long terminal flagellum. The scape (Sc) is collar-shaped and hidden behind the pedicel (Pe), which is a bulbous cup shaped-segment containing Johnston’s organ and provides the attachment of the flagellum. Each flagellum consists of 13 flagellomeres. The surface of the scape, pedicel and first flagellomere is covered densely with aculeae (ac) (Fig. 3a, b). The number of aculeae decreases from the proximal to the distal end of each of flagellomeres 1–5.

### Characterization of antennal sensilla

Like most dipterans, four main types of sensilla occur on the antennae of females of *An. minimus* and *An. harrisoni*, including sensilla chaetica, sensilla trichodea, sensilla basiconica (grooved pegs) and sensilla coeloconica. A fifth type, sensilla ampullacea, occurs on the first flagellomere.

**Fig. 4:**
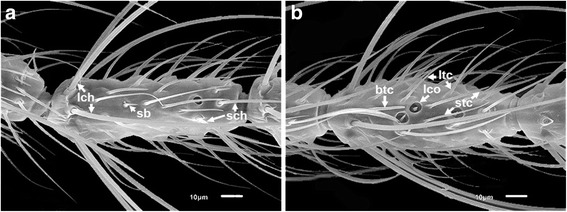
Scanning electron micrographs showing the various types of sensilla borne on the antennae of females of *An. minimus* and *An. harrisoni*. **a** Large sensilla chaetica (lch) with raised sockets; small sensilla chaetica (sch); and a sensillum basiconicum (grooved peg) (sb). **b** Long sharp sensilla trichodea (ltc); short sharp sensilla trichodea (stc); a blunt-tipped sensillum trichodeum (btc); and a large sensillum coeloconicum (lco)

Sensilla chaetica (ch) are long, thick-walled setae set in sturdy sockets (alveoli) and are putative mechanoreceptors [35]. There are large and small subtypes of sensilla chaetica. The shape and distribution of these sensilla are similar in the two species. The large sensilla chaetica (lch) are arranged mostly in a whorl of about six sensilla at the bases of flagellomeres 2–13 (Figs. 2, 3c, 4a). The small sensilla chaetica (sch) usually occur on the distal ends of flagellomeres 2–13 (Fig. 4a). They are interspersed with minute aculeae and their numbers decrease slightly from the proximal to the distal ends of the flagellomeres in both species. Both subtypes also occur on the ventral surface of the first flagellomere and are often interspersed with aculeae (Fig. 3a).

Sensilla trichodea (tc), like sensilla chaetica, are structurally setiform or hair-like sensory structures but they do not arise from a basal alveolus and are olfactory organs [35]. They are the most abundant sensilla on flagellomeres 2–13 of both species. Three subtypes of sensilla trichodea are present: long sharp (acuminate) trichodea (ltc), short sharp (acuminate) trichodea (stc) and blunt-tipped trichodea (btc). Sharp trichodea have a smooth surface (Fig. 4b). The number of long sharp trichodea increases from the proximal to the distal ends of flagellomeres in both species. Short sharp trichodea are fewer in number than the long sharp trichodea on the flagellum of both species (Fig. 4b). Blunt-tipped trichodea, unlike the long sharp trichodea, are not tapered to a point and are more or less of equal diameter throughout their length (Fig. 4b). These sensilla are also apparently more uniform in length than the sharp trichodea. Blunt-tipped sensilla trichodea occur in fewer numbers than the sharp trichodea in both species. However, they do not occur on the first flagellomere.

**Fig. 5:**
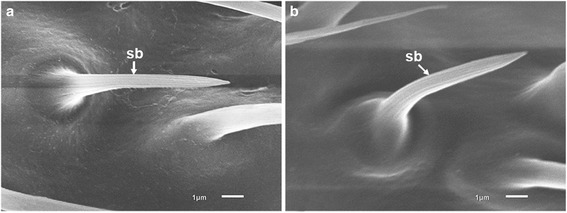
Scanning electron micrographs of sensilla basiconica (sb) on antennae of females of *An. minimus* (**a**) and *An. harrisoni* (**b**)

Sensilla basiconica (sb) are curved peg-like sensilla with approximately 10–12 longitudinal grooves on their surfaces. They arise from slightly raised alveoli (Figs. 4a, 5). Sensilla basiconica are similarly slender, tapered and apically pointed in both *An. minimus* (Fig. 5a) and *An. harrisoni* (Fig. 5b). They are morphological olfactory sensilla [35].

**Fig. 6:**
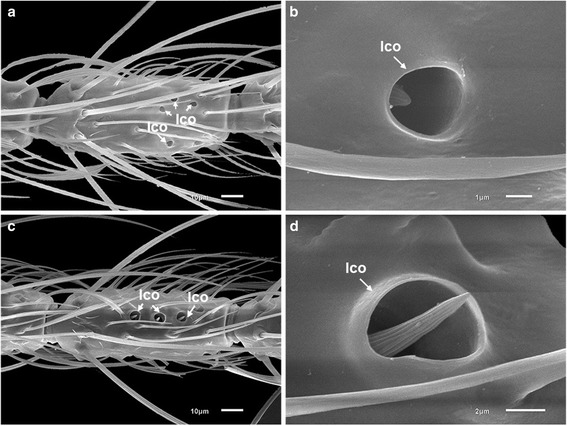
Scanning electron micrographs of two forms of large sensilla coeloconica (lco) observed on antennae of females. **a**, **b** Short form (*An. minimus*), peg not reaching the orifice of the pit. **c**, **d** Long form (*An. harrisoni*), peg extending beyond the orifice of the pit

Sensilla coeloconica (co) are small, thick-walled sensilla that are commonly called pitted pegs because they are borne in a cup-like depression of the antennal wall. The pegs may or may not protrude through the circular openings at the surface of the cuticle. The surface of sensilla coeloconica is grooved lengthwise similar to sensilla basiconica, but the grooves are deeper. They have been putatively classified as hygro- and thermoreceptors [35]. The large sensilla coeloconica (lco) differ on the antennal flagella of *An. minimus* and *An. harrisoni* as follows. Those of *An. minimus* are characterized by short somewhat conical pegs that do not reach the cuticular opening of the pit (Fig. 6a, b). In contrast, the pegs of *An. harrisoni* are much longer and protrude beyond the external rim of the pit (Fig. 6c, d). The distributions of sensilla coeloconica on the antennae of both species based on SEM observation were consistent with the distributions observed using a compound light microscope (Table 1). Small sensilla coeloconica (sco) have the peg set on the bottom of a pit with a small cuticular opening, hence the peg is not visible. This type of sensillum was found on the tip of the distal (13th) antennal flagellomere (Fig. 3c, d).

**Table 1:** Mean distributions of large sensilla coeloconica on antennal flagellomeres 1–8 of adult females of *An. minimus* and *An. harrisoni* (30 females/species, *n* = 60)

Antennal flagellomere (left and right)	Species		*t-*value	*P-*value
	*An. minimus* Mean ± SD (Range)	*An. harrisoni* (range) Mean ± SD (Range)		
1	4.25 ± 1.04 (2–6)	4.73 ± 1.30 (2–8)	2.253	0.026
2	4.55 ± 0.95 (2–6)	5.87 ± 1.13 (3–9)	6.930	< 0.0001
3	4.58 ± 0.85 (2–6)	5.87 ± 1.02 (4–9)	7.505	< 0.0001
4	4.17 ± 0.92 (3–7)	5.30 ± 1.03 (3–8)	6.345	< 0.0001
5	3.83 ± 0.83 (2–6)	4.52 ± 1.11 (2–8)	3.819	< 0.0001
6	2.87 ± 0.57 (2–4)	3.22 ± 0.76 (2–5)	2.857	0.005
7	1.92 ± 0.67 (0–3)	2.22 ± 0.83 (0–4)	2.184	0.031
8	0.02 ± 0.13 (0–1)	0.22 ± 0.49 (0–2)	3.056	0.003
Total (range)	26.25 ± 3.35 (17–35)	31.98 ± 4.71 (22–48)	7.685	< 0.0001

Sensilla ampullacea (sa) are small peg-like organs that are not readily visible because they are borne in pits with narrow or slit-like openings. This type of sensillum occurs on the first antennal flagellomere, surrounded by aculeae (Fig. 3a, b). Sensilla ampullacea are putative hygro- and thermoreceptors [35].

### Number of large sensilla coeloconica on antennae of females

The number of large sensilla coeloconica per antennal flagellomere ranged from 0–7 and 0–9 in *An. minimus* and *An. harrisoni*, respectively. Remarkably, no large sensilla coeloconica were found on flagellomeres 9–13 of either species. The mean number of large sensilla coeloconica on each of flagellomeres 1–8 of the two species was significantly different (Table 1). Likewise, the mean number of large sensilla coeloconica per flagellum of *An. minimus* (26.25) and *An. harrisoni* (31.98) was significantly different (*t*_(118)_ = 7.685, *P* < 0.0001). No surface distribution bias (dorsal and ventral) was observed in either species.

### Mating of mosquitoes in cages of different sizes at DRS 7.2

The mean insemination rates of *An. minimus* were 63 and 55.5 in 20 and 30 cm^3^ cages, respectively. No spermatozoa were found in the spermathecae of *An. harrisoni* placed in cages of these sizes (Table 2). More than 80% of inseminated *An. minimus* females had high sperm density (3+ and 4+) in their spermathecae in cages of both sizes (data not shown).

**Table 2:** Insemination rates of *An. minimus* and *An. harrisoni* females in 20 and 30 cm^3^ cages at Density resting surface (DRS) of 7.2

Species	Mean insemination rate^a^		*P-*value
	20 × 20 × 20 cm	30 × 30 × 30 cm	
*An. minimus*	63	55.5	< 0.0001
*An. harrisoni*	0	0	

^a^Based on dissection of 200 spermathecae/species/cage

### Clasper movement and duration of mating

The frequency of clasper movement in males of the stenogamous *An. minimus* was significantly higher (*t*_(118)_ = -2.984, *P* = 0.030) than in males of *An. harrisoni*. However, the duration of mating of the two species was not significantly different (*t*_(118)_ = -0.889, *P* = 0.370) (Table 3).

**Table 3:** Frequency of clasper movement per copulation and duration of mating (in seconds) measured for *An. harrisoni* and *An. minimus* (*n* = 60)

Species	Frequency of clasper movement Mean ± SD (Range)	*P*-value	Mating time Mean ± SD (Range)	*P*-value
*An. minimus*	10.35 ± 2.62 (5–17)	0.030	9.55 ± 1.56 (7–16)	0.370
*An. harrisoni*	8.98 ± 2.38 (5–16)		9.32 ± 1.29 (6–12)	

## Discussion

Precise species identification of malaria vectors is essential for an understanding of epidemiological patterns of disease transmission and for designing appropriate strategies for vector control. However, it has been impossible to accurately distinguish the two sympatric sibling species, *An. minimus* and *An. harrisoni*, based on larval, pupal and adult morphological characters. During the past couple of decades, several alternative, relatively inexpensive and reliable methods have been reported for mosquito identification. For example, scanning electron microscopy (SEM) revealed various novel characteristics, such as antennal sensilla [36], and features of the cibarial armature [37], eggs [29] and wings [38, 39], which are purportedly useful for identifying species. Somboon et al. [37] reported differences in the cibarial armature of *An. flavirostris*, *An. minimus* and *An. yaeyamaensis* using SEM. However, the antennal sensilla of the two sibling species of the Minimus Complex that occur in Thailand have not been studied before now. The results of the present study show that *An. minimus* and *An. harrisoni* can be distinguished by differences of their antennal sensilla, specifically the form and numbers of sensilla coeloconica on antennal flagellomeres 1–8 (Table 1, Fig. 6).

The antennae of adult mosquitoes bear numerous sensilla, which usually contain one to three receptor neurons (ORNs). In the present study, five types of sensilla with similar distributions were observed on the antennae of females of *An. minimus* and *An. harrisoni*. The same morphological types of sensilla were recognized by Pitts and Zwiebel [36] and Hempolchom et al. [30] in their studies of *Anopheles* mosquitoes, and by Hill et al. [35] in their study of *Culex quinquefasciatus*. Hempolchom et al. [30] constructed a key based on differences in antennal sensilla to reliably distinguish eight species of the *An. hyrcanus* group in Thailand.

It is important to note that the difference in the form of the large sensilla coeloconica present on the antennae of *An. minimus* and *An. harrisoni* (as seen using SEM and noted above) can be used to distinguish and identify them. Additionally, the mean number of large sensilla coeloconica on each flagellum of *An. minimus* (26.25) is significantly fewer than in *An. harrisoni* (31.98). In comparison, the mean number of these sensilla on the antennae of these species is greater than on the antennae of *An. gambiae* (21.6) and *An. quadriannulatus* (29.0) [34] but less than those on the antennae of *An. pursati* (27.85), *An. nitidus* (28.73), *An. nigerrimus* (33.10), *An. sinensis* (36.47) and *An. paraliae* (37.55) [26].

Wijit et al. [26] documented that at least 20 *Anopheles* species are known to be able to successfully copulate in small cages. To shed light on the mechanism(s) that influences the mating behavior of *An. minimus* (stenogamous) and *An. harrisoni* (eurygamous), we compared the insemination rates, the frequency of clasper movement in males during induced copulation and the duration of mating of these species. Unlike *An. minimus*, it appears that females and males of *An. harrisoni* could not copulate in small cages based on the absence of sperm in the spermathecae of females (zero insemination rate). This observation agrees with that of Wijit et al. [26] who reported the highest insemination rates (70–97%) in stenogamous *An. peditaeniatus* in different cage sizes at DRS of 3.6 and 7.2 whereas the eurygamous *An. crawfordi* had the lowest rate (0–4%). The frequency of clasper movement of the stenogamous *An. minimus* was significantly greater than that in the eurygamous *An. harrisoni*; however, the duration of mating of the two species was not significantly different. These results sharply contrast with the finding of Wijit et al. [26], who found that the frequency of clasper movement in the stenogamous *An. peditaeniatus* was lower than that in the seven eurygamous species listed above. Likewise, Sucharit and Choochote [36], working with members of the *An. dirus* complex, found that stenogamous *An. cracens* has a lower frequency of clasper movement and a shorter period of copulation than *An. dirus*.

## Conclusions

Using light and scanning electron microscopy we found that the form and number of large sensilla coeloconica on antennae could distinguish females of *An. minimus* and *An. harrisoni*. The mean number of large sensilla coeloconica on antennal flagellomeres 1–8 (approximately 30 in *An. minimus* and greater than 30 in *An. harrisoni*) should foster the identification of these species. We expect that this discovery will be useful for epidemiological studies in localities where the two species occur in sympatry. It is possible that the faster clasper movement observed in males of *An. minimus* might be an important component of the mechanism that controls the ability of this species to mate in small spaces. The results of the study provide a better understanding of the mating behaviour of both this species and the closely related *An. harrisoni*.

## Abbreviations

ac: Aculeae
AS-PCR: Allele-specific polymerase chain reaction
btc: Blunt-tipped sensilla trichodea
D: Mosquito population density
DRS: Density resting surface
lch: Large sensilla chaetica
lco: Large sensilla coeloconica
ltc: Long sharp sensilla trichodea
Pe: Pedicel
RS: Vertical resting surface area
sa: Sensilla ampullacea
sb: Sensilla basiconica
Sc: Scape
sch: Small sensilla chaetica
sco: Small sensilla coeloconica
SEM: Scanning electron microscopy
stc: Short sharp sensilla trichodea
